# Body Temperature, Heart Rate, and Short-Term Outcome of Cooled Infants

**DOI:** 10.1089/ther.2018.0019

**Published:** 2019-03-06

**Authors:** Kennosuke Tsuda, Sachiko Iwata, Takeo Mukai, Jun Shibasaki, Akihito Takeuchi, Tomoaki Ioroi, Hiroyuki Sano, Nanae Yutaka, Akihito Takahashi, Toshiki Takenouchi, Satoshi Osaga, Takuya Tokuhisa, Sachio Takashima, Hisanori Sobajima, Masanori Tamura, Shigeharu Hosono, Makoto Nabetani, Osuke Iwata

**Affiliations:** ^1^ Center for Human Development and Family Science, Department of Neonatology and Pediatrics, Nagoya City University Graduate School of Medical Sciences, Aichi, Japan.; ^2^ Center for Advanced Medical Research, Institute of Medical Science, University of Tokyo, Tokyo, Japan.; ^3^ Department of Neonatology, Kanagawa Children's Medical Center, Kanagawa, Japan.; ^4^ Division of Neonatology, National Hospital Organization Okayama Medical Center, Okayama, Japan.; ^5^ Department of Pediatrics, Perinatal Medical Center, Himeji Red Cross Hospital, Hyogo, Japan.; ^6^ Department of Pediatrics, Yodogawa Christian Hospital, Osaka, Japan.; ^7^ Department of Pediatrics, Kurashiki Central Hospital, Okayama, Japan.; ^8^ Department of Pediatrics, Keio University School of Medicine, Tokyo, Japan.; ^9^ Clinical Research Management Center, Nagoya City University Hospital, Aichi, Japan.; ^10^ Division of Neonatology, Perinatal Medical Center, Kagoshima City Hospital, Kagoshima, Japan.; ^11^ Yanagawa Institute for Developmental Disabilities, International University of Health and Welfare, Fukuoka, Japan.; ^12^ Division of Neonatology, Center for Maternal, Fetal and Neonatal Medicine, Saitama Medical Center, Saitama Medical University, Saitama, Japan.; ^13^ Department of Pediatrics, Saitama Medical Center, Saitama Medical University, Saitama, Japan.; ^14^ Division of Neonatology, Nihon University Itabashi Hospital, Tokyo, Japan.

**Keywords:** body temperature, heart rate, selective-head cooling, therapeutic hypothermia, whole-body cooling

## Abstract

Therapeutic hypothermia following neonatal encephalopathy is neuroprotective. However, approximately one in two cooled infants still die or develop permanent neurological impairments. Further understanding of variables associated with the effectiveness of cooling is important to improve the therapeutic regimen. To identify clinical factors associated with short-term outcomes of cooled infants, clinical data of 509 cooled infants registered to the Baby Cooling Registry of Japan between 2012 and 2014 were evaluated. Independent variables of death during the initial hospitalization and survival discharge from the cooling hospital at ≤28 days of life were assessed. Death was associated with higher Thompson scores at admission (*p* < 0.001); higher heart rates after 3–72 hours of cooling (*p* < 0.001); and higher body temperature after 24 hours of cooling (*p* = 0.002). Survival discharge was associated with higher 10 minutes Apgar scores (*p* < 0.001); higher blood pH and base excess (both *p* < 0.001); lower Thompson scores (at admission and after 24 hours of cooling; both *p* < 0.001); lower heart rates at initiating cooling (*p* = 0.003) and after 24 hours of cooling (*p* < 0.001) and lower average values after 3–72 hours of cooling (*p* < 0.001); higher body temperature at admission (*p* < 0.001); and lower body temperature after 24 hours and lower mean values after 3–72 hours of cooling (both *p* < 0.001). Survival discharge was best explained by higher blood pH (*p* < 0.05), higher body temperature at admission (*p* < 0.01), and lower body temperature and heart rate after 24 hours of cooling (*p* < 0.01 and <0.001, respectively). Lower heart rate, higher body temperature at admission, and lower body temperature during cooling were associated with favorable short-term outcomes.

## Introduction

Accumulated evidence supports that therapeutic hypothermia following perinatal asphyxia is associated with reduced death and disability up to 18 to 22 months of age (Jacobs *et al.*, 2013). The neuroprotective effect of therapeutic hypothermia is persistently observed up to school age (Guillet *et al.*, 2012; Shankaran *et al.*, 2012; Azzopardi *et al.*, 2014). Subsequently, therapeutic hypothermia has become a routine part of clinical practice for infants with moderate to severe neonatal encephalopathy (Perlman *et al.*, 2015). However, previous large-scale randomized controlled trials demonstrated that 44% to 55% of infants do not respond to therapeutic hypothermia and die or develop permanent disability (Edwards *et al.*, 2010). In addition, recent studies suggest that even infants with mild neonatal encephalopathy who currently have no indication of cooling develop cerebral lesions on MRI and neurodevelopmental impairments thereafter (Rollins *et al.*, 2014; Gagne-Loranger *et al.*, 2016; Walsh *et al.*, 2017).

To improve the therapeutic regimen for neonatal encephalopathy, detailed understanding of clinical variables associated with the effectiveness of therapeutic hypothermia is essential. Recent studies reassessed whether traditional prognostic markers of neonatal encephalopathy are still valid even when cooling is applied. Clinical variables representing the severity of hypoxia-ischemia, such as Apgar scores, blood gas pH and base excess, and encephalopathy scores, showed consistent associations with outcomes in cooled infants (Wyatt *et al.*, 2007; Pappas *et al.*, 2011; Azzopardi *et al.*, 2012), whereas cooling altered the predictive value of several established markers, such as amplitude-integrated encephalogram and cerebral Doppler velocimetry (Takenouchi *et al.*, 2011; Skranes *et al.*, 2014; Del Rio *et al.*, 2016; Chandrasekaran *et al.*, 2017). Apart from traditional markers, several novel independent variables of outcomes have been identified, including body weight and blood glucose and carbon dioxide levels (Wyatt *et al.*, 2007; Pappas *et al.*, 2011; Chouthai *et al.*, 2015; Basu *et al.*, 2017). In contrast, the role of experimentally established independent variables of outcomes, such as delay in cooling and target cooling temperature, has not been confirmed in clinical settings (Gunn *et al.*, 2015; Thoresen, 2015).

This study aimed to identify early clinical factors that may influence short-term outcomes after therapeutic hypothermia in newborn infants with neonatal encephalopathy.

## Materials and Methods

### Ethics approval and consent

This study was conducted in compliance with the Declaration of Helsinki. The protocols of the registry were approved by the Ethics Committees of Kurume University School of Medicine and Saitama Medical University, Japan. Since no patient identifiers were or are collected, the Ethics Committees advised that there is no statutory requirement for parental consent for data collection, and consent was not sought for the current registry.

### Population and data collection

The Baby Cooling Registry of Japan is an online case registry that was established in January 2012 by inviting all registered Japanese level II/III neonatal intensive care centers. The detail of this registry has been reported previously (Tsuda *et al.*, 2017). In brief, participating centers were requested to register all neonates who were referred to the unit for consideration of cooling. Clinical information was provided via the official website, including patient characteristics, severity of encephalopathy, body temperature, cardiovascular/respiratory parameters, supportive treatments, and short-term outcomes. Discharge from the cooling hospital was followed up at least up to 12 months of life. For this observational study, registered data of 509 cooled infants compiled between January 1, 2012 and December 31, 2014 were analyzed.

### Statistical analysis

The Baby Cooling Registry of Japan is currently collecting the follow-up data at 2 years of age, findings of which will be reported elsewhere. For the current study, independent variables of death during the initial hospitalization and survival discharge from the cooling hospital at ≤28 days of life were assessed. Requirements for respiratory and feeding support at discharge (i.e., tube feeding, oxygen supplementation, and other invasive/noninvasive respiratory support) were also assessed. For the analysis, the following 10 clinical background variables were selected: gestational age; birth weight; birth location; 10 minutes Apgar score; cord or first blood pH and base excess; Thompson encephalopathy score at admission and after 24 hours of cooling; elapsed time from birth to the commencement of cooling; and the mode of cooling. Additional 10 physiological variables during cooling were also chosen as potential independent variables of the outcome: body temperature, heart rate, and mean blood pressure at admission (at admission only body temperature was taken) and after 0 and 24 hours and mean values after 3–72 hours of cooling (see Supplementary Table S1 for the analysis involving additional variables; Supplementary Data are available online at www.liebertpub.com/ther). Cases with unexplained missing data for >10% of the aforementioned variables and whose cooling mode and discharge status were not specified were not considered further.

To reduce attrition biases due to missing data, multiple imputation of variables was performed (*n* = 5 imputations) based on the correlation between variables with missing values and other subject characteristics (SPSS ver. 21.0; IBM, Armonk, NY). Univariate logistic regression analysis was performed to evaluate the crude effects of the potential independent variables on the outcome, where statistical significance was assumed for *p*-values <0.005 after correcting for multiple comparisons over 10 variables within the category. Final logistic models to explain the favorable outcome were developed using the independent variables available after 0 and 24 hours of cooling by forward selection.

Subsequently, several novel independent variables of the outcome were identified, including body temperature and heart rate. Independent variables of heart rate (after 0 and 24 hours of cooling) and body temperature (at admission and after 24 hours of cooling) were further investigated using general linear models. Finally, for outborn infants, the association between the target body temperature during transportation and subsequent body temperature at admission was assessed using simple linear regression model.

## Results

### Final study cohort

**FIG. 1.**
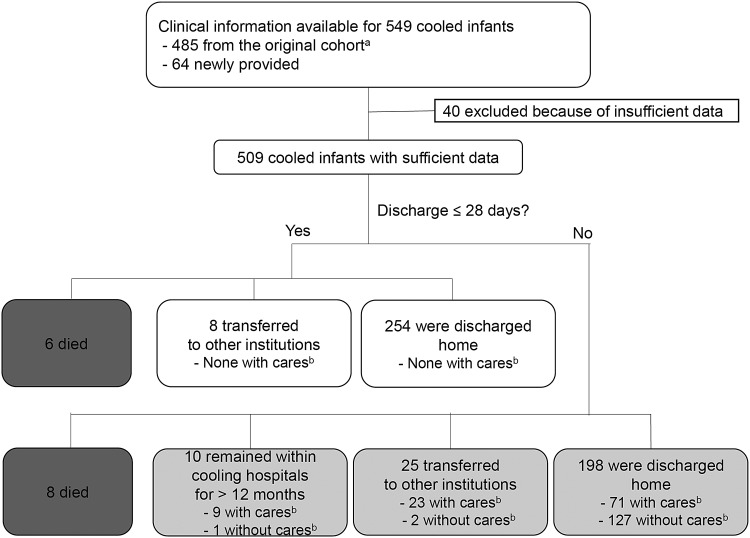
Profile of the study population. ^a^Study cohort used in the previous analysis (see Tsuda *et al.*, 2017 for details). ^b^Continuous medical care, including tube feeding and/or respiratory support (invasive/noninvasive ventilation and oxygen supplementation).

In addition to the data set of 485 cooled infants used in our previous analysis (Tsuda *et al.*, 2017), data were newly obtained for 64 cooled infants, whose data submission was suspended at the time of the previous analysis. Of the 549 infants, 40 did not have sufficient data for the current analysis, and, thus, were excluded. Consequently, the final cohort of the current study comprised 509 cooled newborn infants (Fig. 1). The differences in the study population from that of the original cohort resulted in only subtle changes in the clinical backgrounds, physiological variables, and outcomes of infants (Table 1; see Tsuda *et al.*, 2017 for the outline of the data from the original cohort).

**Table 1.** Independent Variables of Short-term Outcomes

	*Death during initial hospitalization*						*Survival discharge ≤28 days*					
				*95% CI*						*95% CI*		
*Variables*	*Yes (*n* = 14)*	*No (*n* = 495)*	*Odds ratio*	*Lower*	*Upper*	p	*Yes (*n* = 262)*	*No (*n* = 247)*	*Odds ratio*	*Lower*	*Upper*	p
Background variables												
Gestational age (weeks)	38.6 ± 1.8	38.9 ± 1.7	0.876	0.655	1.173	0.374	39.1 ± 1.6	38.7 ± 1.8	1.152	1.040	1.278	0.007
Birth weight (kg)	2.8 ± 0.5	2.9 ± 0.5	0.831	0.274	2.521	0.743	2.9 ± 0.4	2.9 ± 0.5	1.094	0.760	1.576	0.628
Birth location												
Outborn	11 (3.0)	350 (97.0)	1	Reference			174 (48.2)	187 (51.8)	1	Reference		
Inborn	3 (2.0)	145 (98.0)	0.657	0.172	2.502	0.536	88 (59.5)	60 (40.5)	1.576	1.070	2.323	0.021
10 minutes Apgar	2 (1–5)	5 (3–7)	0.705	0.527	0.942	0.020	6 (4–7)	4 (2–5)	1.500	1.362	1.652	**<0.001**
Cord or first blood gas ≤1 hour of birth												
pH	6.85 ± 0.29	6.95 ± 0.21	0.814^a^	0.602	1.101	0.177	6.98 ± 0.18	6.90 ± 0.23	1.212^a^	1.109	1.325	**<0.001**
BE (mmol/L)	−19.8 ± 12.0	−14.5 ± 10.6	0.796^b^	0.442	1.432	0.437	−12.1 ± 9.6	−17.4 ± 11.1	1.575^b^	1.315	1.887	**<0.001**
Thompson encephalopathy score												
At admission	18 (16–19)	10 (7–14)	1.261	1.108	1.434	**<0.001**	9 (6–11)	13 (9–17)	0.862	0.831	0.895	**<0.001**
24 hours^c^	16 (13–18)	10 (5–13)	1.169	1.047	1.304	0.006	7 (3–11)	12 (9–16)	0.865	0.835	0.896	**<0.001**
Cooling modality												
Selective-head	9 (5.1)	167 (94.9)	1	Reference			79 (44.9)	97 (55.1)	1	Reference		
Whole-body	5 (1.5)	327 (98.5)	0.343	0.108	1.089	0.069	182 (54.8)	150 (45.2)	1.498	1.038	2.163	0.031
Initiating cooling after birth (minutes)	233 ± 104	212 ± 96	1.034^d^	0.979	1.093	0.229	214 ± 95	211 ± 97	1.003^d^	0.985	1.022	0.730
Physiological variables during cooling												
Heart rate (beat/min)												
0 hour^c^	142 ± 21	132 ± 20	1.226^e^	0.957	1.570	0.107	129 ± 20	135 ± 19	0.857^e^	0.777	0.946	**0.003**
24 hours^c^	133 ± 14	114 ± 18	1.777^e^	1.206	2.618	0.005	108 ± 17	121 ± 17	0.644^e^	0.563	0.737	**<0.001**
Mean (3–72 hours^c^)	133 ± 14	113 ± 14	2.595^e^	1.749	3.850	**<0.001**	106 ± 12	120 ± 14	0.429^e^	0.354	0.519	**<0.001**
Mean blood pressure (mmHg)												
0 hour^c^	41 ± 12	46 ± 10	0.588^f^	0.347	0.995	0.048	47 ± 9	46 ± 11	1.103^f^	0.924	1.317	0.276
24 hours^c^	41 ± 10	47 ± 8	0.306^f^	0.118	0.792	0.017	47 ± 7	47 ± 8	1.012^f^	0.807	1.268	0.921
Mean (3–72 hours^c^)	42 ± 10	49 ± 6	0.295^f^	0.083	1.051	0.059	48 ± 5	48 ± 7	0.854^f^	0.618	1.180	0.339
Body temperature (°C)												
At admission	35.2 ± 1.0	36.0 ± 1.3	0.653	0.463	0.922	0.016	36.2 ± 1.1	35.7 ± 1.4	1.372	1.157	1.627	**<0.001**
0 hour^c^	34.5 ± 1.3	35.3 ± 1.3	0.639	0.425	0.960	0.031	35.4 ± 1.2	35.1 ± 1.3	1.176	1.010	1.369	0.037
24 hours^c^	34.2 ± 1.2	33.8 ± 0.5	3.558	1.631	7.761	**0.002**	33.7 ± 0.5	33.9 ± 0.5	0.459	0.316	0.666	**<0.001**
Mean (3–72 hours^c^)	34.1 ± 0.7	33.8 ± 0.5	2.268	1.063	4.842	0.034	33.7 ± 0.5	33.9 ± 0.5	0.404	0.269	0.606	**<0.001**

Values are shown as number (%), mean ± standard deviation, or median (interquartile range).

Statistical significance was assumed for *p* < 0.005 (indicated in bold, Bonferroni correction).

^a^ Per 0.1 change.

^b^ Per 10 mmol/L.

^c^ After initiating cooling.

^d^ Per 10 minutes.

^e^ Per 10 beat/min.

^f^ Per 10 mmHg.

BE, base excess; CI, confidence interval.

### Outcome of infants

By the 28th day of life, 6 infants died during the initial hospitalization, 8 infants were transferred to a birth hospital or neighboring institutions, and 254 infants were discharged home (none required respiratory/feeding support; Fig. 1). Of the remaining 241 infants who required hospital care beyond 28 days of life, 8 infants died during the initial hospital stay, 10 infants remained hospitalized within the cooling unit at 12 months of life, 25 infants were transferred to other institutions, and 198 infants were discharged home.

### Independent variables of death during initial hospitalization

**FIG. 2.**
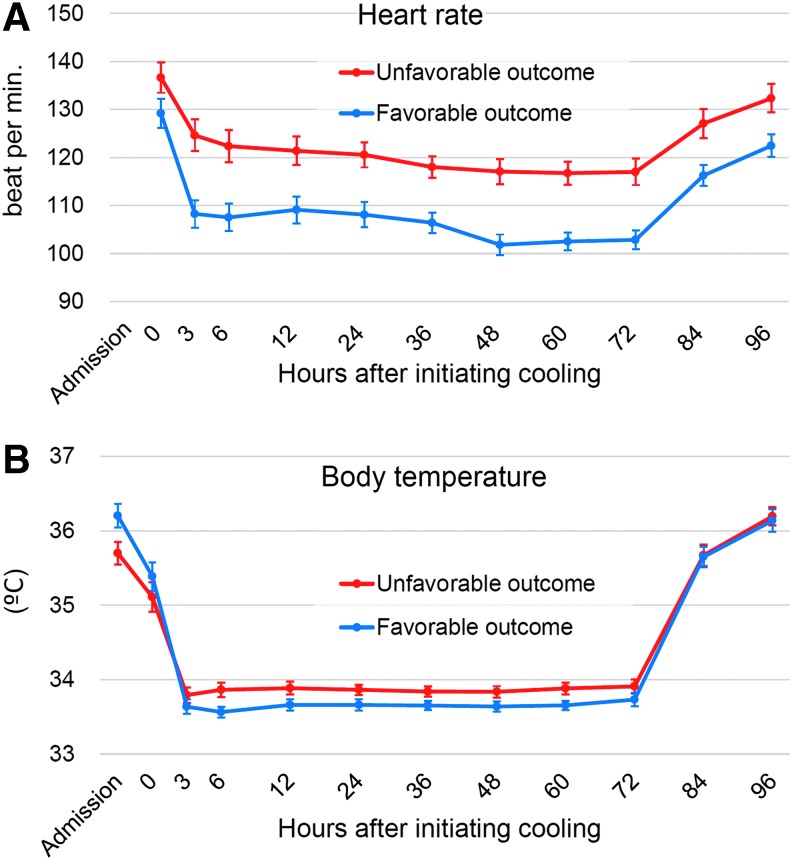
Temporal changes of heart rate **(A)** and body temperature **(B)** in infants with favorable and unfavorable outcomes. Values are shown as mean (95% confidence interval). Data at admission were not collected for the heart rate.

Death during initial hospitalization was associated with higher Thompson encephalopathy scores at admission (*p* <0.001); higher heart rates after 3–72 hours of cooling (*p* < 0.001); and higher body temperature after 24 hours of cooling (*p* = 0.002) (Table 1, Fig. 2, and Supplementary Table S1). Multivariate analysis was not performed because of the small number of mortality events. Potential dependence of the outcome on 10 minutes Apgar scores; Thompson encephalopathy scores after 24 hours of cooling; heart rates after 24 hours of cooling; mean blood pressure at initiating cooling and after 24 hours of cooling; and body temperature at admission, at initiating cooling; and mean values after 3–72 hours of cooling was lost after correction for multiple comparisons.

### Independent variables of survival discharge within 28 days of life

Survival discharge ≤28 days was associated with higher 10 minutes Apgar scores (*p* < 0.001); higher blood pH and base excess (both *p* < 0.001); lower Thompson encephalopathy scores (at admission and after 24 hours of cooling; both *p* < 0.001); lower heart rates at initiating cooling (*p* = 0.003) and after 24 hours of cooling (*p* < 0.001); and lower mean values after 3–72 hours of cooling (*p* < 0.001); higher body temperature at admission (*p* < 0.001); and lower body temperature after 24 hours and lower mean values after 3–72 hours of cooling (both *p* < 0.001) (Table 1, Fig. 2, and Supplementary Table S1). Potential dependence of the outcome on greater gestational age, inborn, whole-body cooling, and higher body temperature at initiating cooling was lost after correction for multiple comparisons.

The multivariate logistic regression model to estimate survival discharge ≤28 days at the time of cooling initiation comprised inborn (*p* < 0.05), greater gestational age (*p* < 0.05), higher blood gas pH (*p* < 0.005), lower heart rate at initiation of cooling (*p* < 0.001), and higher body temperature at admission (*p* < 0.001) (Table 2). When clinical variables obtained up to 24 hours after initiation of cooling were incorporated, the final model to predict survival discharge ≤28 days consisted of higher blood gas pH (*p* < 0.05), higher body temperature at admission (*p* < 0.01), lower body temperature after 24 hours of cooling (*p* < 0.01), and lower heart rate after 24 hours of cooling (*p* < 0.001) (Table 3).

**Table 2.** Multivariate Model Using Variables Available at the Commencement of Cooling

		*95% CI*		
	*Odds ratio*	*Lower*	*Upper*	p
Birth location (inborn)	1.604	1.049	2.453	**0.029**
Gestational age (per week)	1.140	1.019	1.276	**0.022**
Cord or first blood gas pH (per 0.1 change)	1.148	1.046	1.258	**0.003**
Heart rate at 0 hour^a^ (per 10 beat/min)	0.824	0.741	0.917	**<0.001**
Body temperature at admission (per degree)	1.364	1.149	1.620	**<0.001**

Statistical significance was assumed for *p* < 0.05 (indicated in bold).

^a^ After initiating cooling.

**Table 3.** Multivariate Model Using Variables Available After 24 Hours of Cooling

		*95% CI*		
	*Odds ratio*	*Lower*	*Upper*	p
Cord or first blood gas pH (per 0.1 change)	1.112	1.006	1.228	**0.037**
Body temperature at admission (per degree)	1.271	1.076	1.503	**0.005**
Heart rate at 24 hours^a^ (per 10 beat/min)	0.689	0.594	0.799	**<0.001**
Body temperature at 24 hours^a^ (per degree)	0.558	0.372	0.838	**0.005**

^a^ After initiating cooling. Statistical significance was assumed for *p* < 0.05 (indicated in bold).

### Control variables of heart rate and body temperature

Higher heart rate at initiation of cooling was associated with lower blood base excess (*p* < 0.01), higher Thompson encephalopathy scores at admission (*p* < 0.01), and higher body temperature at admission (*p* < 0.001) (Supplementary Table S2). The higher heart rate after 24 hours of cooling could be best explained by the lower first blood base excess, higher Thompson encephalopathy scores at admission, and higher body temperature after 24 hours of cooling (all *p* < 0.001) (Supplementary Table S3). Higher body temperature at admission was associated with inborn (*p* < 0.01), greater birth weight (*p* < 0.001), higher first blood base excess (*p* < 0.005), and smaller Thompson encephalopathy scores (*p* < 0.05) (Supplementary Table S4). Higher body temperature after 24 hours of cooling was associated with lower 10 minutes Apgar score (*p* < 0.01) and selective-head cooling (*p* < 0.001). In a cohort of outborn infants, the body temperature at admission was dependent on the target body temperature during transportation (*p* < 0.001, *r* = 0.394) (Supplementary Table S5).

## Discussion

Using a large-scale data set from a national registry, we identified a range of potentially important clinical variables associated with outcomes of cooled infants. Outborn, younger gestational age, and higher heart rate before, during, and after cooling were associated with adverse outcomes. Higher body temperature at admission and lower body temperature during cooling were paradoxically associated with survival discharge ≤28 days.

### Heart rate and outcome

Bradycardia is a major physiological response to hypothermia (Thoresen *et al.*, 2000; Erecinska *et al.*, 2003). Studies of out-of-hospital cardiac arrest suggested that sinus bradycardia of <50–60 beat/min under cooling is associated with favorable outcomes (Staer-Jensen *et al.*, 2014; Thomsen *et al.*, 2015). Elstad et al. (2016) found in 60 cooled newborn infants that poor outcome is associated with higher heart rate after 12 hours of birth. In a large cohort of newborn infants, we confirmed that higher heart rates before, during, and after cooling are consistently associated with adverse outcome. The precise mechanism remains unclear, however, following severe hypoxia–ischemia, sympathetic stimulation due to excessive release of excitatory neurotransmitters may impair autoregulation of the cardiac system (Drury *et al.*, 2014; Govindan *et al.*, 2016). Restricted cardiac output and relatively higher body temperature may also be a common causative of tachycardia and adverse outcomes. However, in our data, tachycardia was not associated with hypotension; the relationship between tachycardia and adverse outcomes was observed even when corrected for the influence of body temperature. Although we did not obtain information on the use of inotropic and sedative drugs, it is possible that these therapeutic options were predominantly used for relatively more severely asphyxiated infants, resulting in a spurious correlation between tachycardia and adverse outcomes. Further studies are required to investigate the interactions between body temperature, heart rate, and outcome by incorporating various clinical variables, including cardiac support.

### Body temperature and outcome

Preclinical studies consistently demonstrated the dependence of the outcome on the timing of cooling initiation (Davidson *et al.*, 2015; Thoresen, 2015). However, this has not been confirmed in clinical studies. In addition, unlike induced hypothermia, spontaneous temperature reduction before initiating cooling might have a different influence on the outcome. Spontaneous hypothermia following severe hypoxia–ischemia or behavioral hypothermia has been observed in numerous vertebrates (Wood *et al.*, 1996). Using a postnatal day 7 rat pup model of hypoxia–ischemia, Wood *et al.* (2017) demonstrated that spontaneous body temperature reduction of <32.2°C 1 hour after hypoxia–ischemia is associated with more severe brain injury, whereas active cooling to 32°C improved histopathological brain injury compared with normothermic temperature management. Consistent with these findings, in our study population, lower body temperature at admission and higher body temperature during cooling were paradoxically associated with adverse outcomes. Several explanations are possible. Severe hypoxia–ischemia may trigger self-protection programs to downregulate intrinsic thermogenesis (Wood *et al.*, 1996). Profound brain stem injury may affect the thermoregulatory response to heat loss (George *et al.*, 2004). Our data suggested that lower body temperature at admission was associated with outborn, whereas the body temperature in outborn infants was dependent on the target body temperature during transportation. It is possible that the initiation of cooling before receiving intensive care is deleterious under certain circumstances.

Regarding the cooling temperature, experimental studies suggested that even subtle temperature differences may alter the neuroprotective effect of hypothermia (Leonov *et al.*, 1990; Iwata *et al.*, 2005; Wood *et al.*, 2017); however, this trend has not been confirmed in the clinical setting. Although a recent large-scale trial did not find any additional benefits of cooling newborn infants to 32°C (Shankaran *et al.*, 2017), our data suggested the potential advantage of using relatively lower cooling temperatures within the currently recommended range. In our study, slightly higher temperature levels during cooling were primarily associated with selective-head cooling, which uses 1°C-higher body temperature than whole-body cooling. Hoque et al. (2010) also reported that the fluctuation of the rectal temperature was greater for selective-head cooling compared with whole-body cooling. Potential advantages in using whole-body cooling need to be investigated.

### Other independent variables of outcome

Our data confirmed the dependence of outcomes of cooled infants on established markers for the severity of hypoxia–ischemia and encephalopathy (Sarnat *et al.*, 1976; Thompson *et al.*, 1997; van de Riet *et al.*, 1999; Malin *et al.*, 2010). In addition, outborn and younger gestational age were identified as independent variables of adverse outcomes. Rao et al. (2017) assessed the safety of cooling preterm infants (34–35 weeks' gestation), compared the results with those of term infants, and found consistent trends toward increased adverse events, such as hypo/hyperglycemia, MRI brain lesions, and death. In our study cohort, the dependence of outcomes on gestational age was consistently observed even when the analysis was repeated after excluding 18 infants <36 weeks gestation (data not shown). A special consideration would be necessary in cooling infants with a relatively younger gestational age.

### Limitations

Because of the revision of the national guideline for the handling of the clinical data in 2017, the Baby Cooling Registry of Japan is currently suspending the data collection. Subsequently, the outcome was assessed using short-term endpoints. Furthermore, multivariate models were developed only for survival discharge ≤28 days due to the low mortality rate. The timing of discharge is affected by subjective decisions. However, we rarely encounter near-term and term infants requiring prolonged hospitalization >28 days unless they have serious respiratory/feeding problems. Indeed, of 198 infants who were discharged home after 28 days, 35.9% were dependent on continuous respiratory support and/or enteral feeding, as opposed to none requiring medical care for ones discharged ≤28 days. We previously speculated that a Japanese cultural background, where withdrawal from life support is relatively uncommon, is at least, in part, responsible for the low mortality rate of cooled infants in our registry (Tsuda *et al.*, 2017). Therefore, careful consideration is required when interpreting our current findings into clinical practice in other part of the world.

Because of these limitations, the precise relationships between the clinical variables and outcomes largely remain unknown. Nonetheless, the use of short-term measures would be justified when the safety of specific cooling procedures is concerned (Gunn *et al.*, 1998; Thoresen *et al.*, 2000; Shankaran *et al.*, 2014; Rao *et al.*, 2017). We believe that clinicians and researchers, who wish to improve the outcome of infants with neonatal encephalopathy, should be aware of our preliminary findings, especially of the possible risk of relatively lower body temperature at admission and relatively higher body temperature during cooling in some specific conditions.

## Conclusions

In addition to the established outcome markers of cooled infants, greater gestational age, inborn, higher body temperature at admission, lower body temperature during cooling, and bradycardia before, during, and after cooling were identified as potential independent variables of favorable short-term outcomes. With further investigations, these novel variables may help improve the therapeutic regimen for neonatal encephalopathy by (1) uncovering new mechanisms (and therapeutic target) of brain injury, (2) improving the algorithm of outcome prediction, and (3) renewing the criteria for patient selection. Prospective studies need to investigate the cause-consequence relationships between body temperature, heart rate, and outcomes. Meanwhile, secondary analyses of pooled data from previous large-scale trials should be conducted.

## Supplementary Material

Supplemental data

Supplemental data

Supplemental data

Supplemental data

Supplemental data
