# Supplementary material for: Body Temperature, Heart Rate, and Short-Term Outcome of Cooled Infants
Source: Ther Hypothermia Temp Manag. 2019 Mar 6;9(1):76–85. doi: 10.1089/ther.2018.0019 (PMC6434598; doi:10.1089/ther.2018.0019)
Supplement: Supplemental data [file Supp_Table1.pdf]

Supplementary Data

Supplementary Table S1. Full Independent Variables of Short-term Outcomes

	Death during initial hospitalization			95% CI		
Background clinical variables	*Yes* (n=*14*)	*No* (n=*495*)	*OR*	*Lower*	*Upper*	p
(A) Full independent variables of the death during initial hospitalization						
Year of birth						
2012	7 (3.7)	180 (96.3)	1	Reference		
2013	3 (1.9)	158 (98.1)	0.647	0.172	2.427	0.518
2014	4 (2.5)	157 (97.5)	0.781	0.223	2.731	0.698
Gestational age (weeks)	38.6±1.8	38.9±1.7	0.876	0.655	1.173	0.374
Birth weight (kg)	2.8±0.5	2.9±0.5	0.831	0.274	2.521	0.743
Birth location						
Outborn	11 (3.0)	350 (97.0)	1	Reference		
Inborn	3 (2.0)	145 (98.0)	0.657	0.172	2.502	0.536
10 minutes Apgar score	2 (1–5)	5 (3–7)	0.705	0.527	0.942	0.020
Cord or first blood gas <1 hour of birth						
pH	6.85±0.29	6.95±0.21	0.814[a]	0.602	1.101	0.177
Base excess (mmol/L)	−19.8±12.0	−14.5±10.6	0.796[b]	0.442	1.432	0.437
Sarnat encephalopathy stage at admission						
Stage I	0 (0.0)	61 (100.0)		Not applicable		
Stage II	4 (1.3)	298 (98.7)	1	Reference		
Stage III	10 (7.6)	122 (92.4)	7.394	2.307	23.704	0.001
Thompson encephalopathy score						
At admission	18 (16–19)	10 (7–14)	1.261	1.108	1.434	<0.001
24 hours[c]	16 (13–18)	10 (5–13)	1.169	1.047	1.304	0.006
48 hours[c]	16 (11–17)	9 (3–13)	1.145	1.033	1.269	0.010
72 hours[c]	15 (0–18)	6 (0–12)	1.111	1.015	1.215	0.022
Maximum score	18 (16–19)	11 (8–15)	1.267	1.099	1.461	0.001
Cooling modality						
Selective-head	9 (5.1)	167 (94.9)	1	Reference		
Whole-body	5 (1.5)	327 (98.5)	0.343	0.108	1.089	0.069
Additive neuroprotective drug						
Magnesium sulfate	5 (4.0)	121 (96.0)	1.427	0.478	4.263	0.524
Erythropoietin	0 (0.0)	17 (100.0)		Not applicable		
Phenobarbital	0 (0.0)	5 (100.0)		Not applicable		
Any of above	5 (1.5)	155 (96.9)	0.979	0.330	2.907	0.970
Time of admission after birth[a]	112±90	107±83	1.020[d]	0.964	1.081	0.488
Initiating cooling after admission[a]	121±103	105±86	1.017[d]	0.958	1.080	0.584
Initiating cooling after birth[a]	233±104	212±96	1.034[d]	0.979	1.093	0.229
Time to target temperature after initiating cooling[a]	45±106	95±155	0.961[d]	0.883	1.047	0.355
Time to target temperature after birth[a]	292±165	307±188	0.998[d]	0.968	1.028	0.888
Physiological variables during cooling						
Heart rate (per 10 beat/min)						
0 hour[c]	142±21	132±20	1.226[e]	0.957	1.570	0.107
3 hours[c]	138±17	116±20	1.550[e]	1.201	2.001	0.001
6 hours[c]	138±19	114±20	1.639[e]	1.227	2.190	0.001
12 hours[c]	135±13	115±19	1.603[e]	1.211	2.120	0.001
24 hours[c]	133±14	114±18	1.777[e]	1.206	2.618	0.005
36 hours[c]	139±16	112±16	2.336[e]	1.633	3.343	<0.001
48 hours[c]	131±11	109±17	2.124[e]	1.485	3.038	<0.001
60 hours[c]	127±10	109±16	1.782[e]	1.315	2.416	<0.001
72 hours[c]	125±19	109±16	1.711[e]	1.208	2.423	0.004
84 hours[c]	138±23	121±19	1.428[e]	0.831	2.452	0.166
Mean (0–84 hours[c])	135±14	115±14	2.589[e]	1.708	3.922	<0.001
Mean (3–72 hours[c])	133±14	113±14	2.595[e]	1.749	3.850	<0.001
Mean blood pressure (per 10 mmHg)						
0 hour[c]	41±12	46±10	0.588[f]	0.347	0.995	0.048
3 hours[c]	39±10	47±9	0.407[f]	0.222	0.744	0.004
6 hours[c]	41±12	47±9	0.475[f]	0.240	0.941	0.033
12 hours[c]	41±14	47±8	0.424[f]	0.215	0.835	0.013

(*continued*)

Supplementary Table S1. (Continued)

Background clinical variables	Death during initial hospitalization: Yes (n = 14)	No (n = 495)	OR	95% CI Lower	Upper	p
24 hours[c]	41 ± 10	47 ± 8	0.306[f]	0.118	0.792	0.017
36 hours[c]	48 ± 9	48 ± 7	0.859[f]	0.347	2.128	0.733
48 hours[c]	53 ± 9	49 ± 7	1.494[f]	0.710	3.144	0.285
60 hours[c]	53 ± 11	50 ± 8	1.013[f]	0.305	3.365	0.981
72 hours[c]	49 ± 6	52 ± 8	0.581[f]	0.193	1.747	0.304
84 hours[c]	52 ± 4	50 ± 8	0.927[f]	0.416	2.064	0.845
Mean (0–84 hours[c])	42 ± 10	48 ± 5	0.297[f]	0.090	0.976	0.046
Mean (3–72 hours[c])	42 ± 10	49 ± 6	0.295[f]	0.083	1.051	0.059
Body temperature (per degree)						
At admission	35.2 ± 1.0	36.0 ± 1.3	0.653	0.463	0.922	0.016
0 hour[c]	34.5 ± 1.3	35.3 ± 1.3	0.639	0.425	0.960	0.031
3 hours[c]	34.0 ± 0.7	33.8 ± 0.7	1.360	0.719	2.573	0.344
6 hours[c]	34.2 ± 0.7	33.8 ± 0.6	2.110	1.126	3.954	0.020
12 hours[c]	34.0 ± 0.7	33.8 ± 0.6	1.439	0.675	3.069	0.346
24 hours[c]	34.2 ± 1.2	33.8 ± 0.5	3.558	1.631	7.761	0.002
36 hours[c]	34.1 ± 0.9	33.8 ± 0.5	4.097	1.821	9.218	0.001
48 hours[c]	33.7 ± 0.4	33.8 ± 0.5	2.250	1.009	5.017	0.047
60 hours[c]	33.8 ± 0.4	33.8 ± 0.5	2.226	1.055	4.693	0.036
72 hours[c]	33.8 ± 0.4	33.8 ± 0.6	1.795	0.767	4.199	0.170
84 hours[c]	34.9 ± 1.0	35.7 ± 1.0	0.974	0.399	2.374	0.947
Mean (0–84 hours[c])	34.2 ± 0.7	34.1 ± 0.4	2.179	0.715	6.635	0.168
Mean (3–72 hours[c])	34.1 ± 0.7	33.8 ± 0.5	2.268	1.063	4.842	0.034
Fever ≥37.5°C from admission to 84 hours[c]	0 (0.0)	50 (100.0)		Not applicable		
Fever ≥38°C from admission to 84 hours[c]	0 (0.0)	18 (100.0)				

[a]Per 0.1 change.
[b]Per 10 mmol/L.
[c]After initiating cooling.
[d]Per 10 minutes.
[e]Per 10 beat/min.
[f]Per 10 mmHg.

Background clinical variables	Survival discharge ≤28 days: Yes (n = 262)	No (n = 247)	OR	95% CI Lower	Upper	p
(B) Full independent variables of survival discharge within 28 days of life						
Year of birth						
2012	88 (47.1)	99 (52.9)	1	Reference		
2013	83 (51.6)	78 (48.4)	1.197	0.785	1.825	0.403
2014	91 (56.5)	70 (43.5)	1.463	0.957	2.234	0.079
Gestational age (weeks)	39.1 ± 1.6	38.7 ± 1.8	1.152	1.040	1.278	0.007
Birth weight (kg)	2.9 ± 0.4	2.9 ± 0.5	1.094	0.760	1.576	0.628
Birth location						
Outborn	174 (48.2)	187 (51.8)	1	Reference		
Inborn	88 (59.5)	60 (40.5)	1.576	1.070	2.323	0.021
10 minutes Apgar score	6 (4–7)	4 (2–5)	1.500	1.362	1.652	<0.001
Cord or first blood gas <1 hour of birth						
pH	6.98 ± 0.18	6.90 ± 0.23	1.212[a]	1.109	1.325	<0.001
Base excess (mmol/L)	−12.1 ± 9.6	−17.4 ± 11.1	1.575[b]	1.315	1.887	<0.001
Sarnat encephalopathy stage at admission						
Stage I	45 (73.8)	16 (26.2)	1	Reference		
Stage II	193 (63.9)	109 (36.1)	0.617	0.332	1.147	0.127
Stage III	14 (10.6)	118 (89.4)	0.042	0.018	0.094	<0.001
Thompson encephalopathy score						
At admission	9 (6–11)	13 (9–17)	0.862	0.831	0.895	<0.001
24 hours[c]	7 (3–11)	12 (9–16)	0.865	0.835	0.896	<0.001
48 hours[c]	7 (2–10)	12 (7–15)	0.872	0.843	0.903	<0.001
72 hours[c]	4 (0–8)	10 (3–15)	0.868	0.839	0.898	<0.001
Maximum score	9 (6–12)	14 (11–17)	0.848	0.815	0.883	<0.001
Cooling modality						
Selective-head	79 (44.9)	97 (55.1)	1	Reference		
Whole-body	182 (54.8)	150 (45.2)	1.498	1.038	2.163	0.031

(*continued*)

Supplementary Table S1. (Continued)

Background clinical variables	Survival discharge ≤28 days			95% CI		
	Yes (n = 262)	No (n = 247)	OR	Lower	Upper	p
Additive neuroprotective drug						
Magnesium sulfate	67 (53.2)	59 (46.8)	1.095	0.732	1.639	0.660
Erythropoietin	9 (52.9)	8 (47.1)	1.063	0.403	2.800	0.902
Phenobarbital	1 (20.0)	4 (80.0)	0.233	0.026	2.097	0.194
Any of above	82 (51.3)	78 (48.8)	0.985	0.677	1.434	0.938
Time of admission after birth[a]	105 ± 86	109 ± 80	0.993[d]	0.971	1.014	0.498
Initiating cooling after admission[a]	109 ± 86	102 ± 87	1.012[d]	0.990	1.035	0.286
Initiating cooling after birth[a]	214 ± 95	211 ± 97	1.003[d]	0.985	1.022	0.730
Time to target temperature after initiating cooling[a]	105 ± 183	82 ± 116	1.009[d]	0.996	1.021	0.174
Time to target temperature after birth[a]	320 ± 208	292 ± 162	1.007[d]	0.997	1.016	0.186
Physiological variables during cooling						
Heart rate (per 10 beat/min)						
0 hour[c]	129 ± 20	135 ± 19	0.857[e]	0.777	0.946	0.003
3 hours[c]	109 ± 18	124 ± 20	0.668[e]	0.599	0.745	<0.001
6 hours[c]	108 ± 19	123 ± 20	0.689[e]	0.614	0.773	<0.001
12 hours[c]	109 ± 18	122 ± 18	0.690[e]	0.613	0.777	<0.001
24 hours[c]	108 ± 17	121 ± 17	0.644[e]	0.563	0.737	<0.001
36 hours[c]	106 ± 15	119 ± 16	0.608[e]	0.529	0.699	<0.001
48 hours[c]	103 ± 14	117 ± 16	0.522[e]	0.449	0.607	<0.001
60 hours[c]	103 ± 13	117 ± 17	0.530[e]	0.456	0.616	<0.001
72 hours[c]	103 ± 13	117 ± 17	0.499[e]	0.422	0.590	<0.001
84 hours[c]	116 ± 16	127 ± 21	0.732[e]	0.660	0.811	<0.001
Mean (0–84 hours[c])	109 ± 11	123 ± 13	0.409[e]	0.335	0.500	<0.001
Mean (3–72 hours[c])	106 ± 12	120 ± 14	0.429[e]	0.354	0.519	<0.001
Mean blood pressure (per 10 mmHg)						
0 hour[c]	47 ± 9	46 ± 11	1.103[f]	0.924	1.317	0.276
3 hours[c]	47 ± 8	47 ± 10	0.998[f]	0.820	1.215	0.985
6 hours[c]	47 ± 8	47 ± 9	1.052[f]	0.858	1.289	0.625
12 hours[c]	48 ± 8	46 ± 9	1.229[f]	0.985	1.534	0.068
24 hours[c]	47 ± 7	47 ± 8	1.012[f]	0.807	1.268	0.921
36 hours[c]	48 ± 7	49 ± 8	0.891[f]	0.693	1.145	0.366
48 hours[c]	49 ± 6	50 ± 8	0.793[f]	0.613	1.025	0.076
60 hours[c]	49 ± 7	52 ± 9	0.694[f]	0.551	0.873	0.002
72 hours[c]	51 ± 8	52 ± 9	0.830[f]	0.671	1.026	0.085
84 hours[c]	49 ± 8	51 ± 9	0.868[f]	0.691	1.092	0.222
Mean (0–84 hours[c])	48 ± 5	48 ± 7	0.872[f]	0.626	1.215	0.418
Mean (3–72 hours[c])	48 ± 5	48 ± 7	0.854[f]	0.618	1.180	0.339
Body temperature (per degree)						
At admission	36.2 ± 1.1	35.7 ± 1.4	1.372	1.157	1.627	<0.001
0 hour[c]	35.4 ± 1.2	35.1 ± 1.3	1.176	1.010	1.369	0.037
3 hours[c]	33.7 ± 0.7	33.8 ± 0.7	0.774	0.600	0.999	0.049
6 hours[c]	33.7 ± 0.6	33.9 ± 0.6	0.510	0.370	0.703	<0.001
12 hours[c]	33.7 ± 0.6	33.9 ± 0.6	0.588	0.424	0.815	0.001
24 hours[c]	33.7 ± 0.5	33.9 ± 0.5	0.459	0.316	0.666	<0.001
36 hours[c]	33.7 ± 0.5	33.9 ± 0.5	0.460	0.316	0.670	<0.001
48 hours[c]	33.7 ± 0.4	33.9 ± 0.5	0.440	0.302	0.639	<0.001
60 hours[c]	33.7 ± 0.5	33.9 ± 0.6	0.446	0.314	0.633	<0.001
72 hours[c]	33.8 ± 0.6	33.9 ± 0.6	0.610	0.452	0.823	0.001
84 hours[c]	35.6 ± 1.0	35.7 ± 1.0	1.016	0.775	1.331	0.902
Mean (0–84 hours[c])	34.1 ± 0.5	34.2 ± 0.4	0.517	0.330	0.809	0.004
Mean (3–72 hours[c])	33.7 ± 0.5	33.9 ± 0.5	0.404	0.269	0.606	<0.001
Fever ≥37.5°C from admission to 84 hours[c]	28 (51.3)	22 (44.0)	1.188	0.667	2.116	0.558
Fever ≥38°C from admission to 84 hours[c]	9 (50.0)	9 (50.0)	0.975	0.391	2.431	0.957

[a]Per 0.1 change.
[b]Per 10 mmol/L.
[c]After initiating cooling.
[d]Per 10 minutes.
[e]Per 10 beat/min.
[f]Per 10 mmHg.
BE, base excess; bpm, beat per minutes; CI, confidence interval; OR, odds ratio.
