# Supplementary material for: Body Temperature, Heart Rate, and Short-Term Outcome of Cooled Infants
Source: Ther Hypothermia Temp Manag. 2019 Mar 6;9(1):76–85. doi: 10.1089/ther.2018.0019 (PMC6434598; doi:10.1089/ther.2018.0019)
Supplement: Supplemental data [file Supp_Table2.pdf]

Supplementary Table S2. Control Variables of Heart rate After 0 Hour of Cooling

	Regression coefficient	*95% CI*		p
		Lower	*Upper*	
(A) Univariate analysis				
Gestational age (weeks)	0.145	–0.954	1.244	0.796
Birth weight (kg)	1.773	–2.166	5.712	0.377
Birth location (outborn)	1.536	–2.614	5.685	0.467
10 minutes Apgar score	–0.913	–1.919	0.093	0.073
Cord or first blood gas pH (per 0.1 change)	–0.732	–1.670	0.207	0.125
Cord or first blood gas base excess (per 10 mmol/L)	–2.090	–3.947	–0.233	0.028
Time of admission after birth[a]	0.193	–0.033	0.420	0.094
Initiating cooling after admission[a]	–0.213	–0.445	0.020	0.072
Initiating cooling after birth[a]	–0.001	–0.192	0.190	0.993
Sarnat encephalopathy stage at admission	7.177	4.183	10.170	**<0.001**
Thompson encephalopathy score at admission	0.424	0.094	0.753	0.012
Mean blood pressure at 0 hour[b] (per 10 mmHg)	1.432	–0.707	3.570	0.185
Body temperature at admission (°C)	2.905	1.001	4.810	0.005
Body temperature at 0 hour[b] (°C)	5.573	3.970	7.175	**<0.001**
(B) Multivariate model				
Cord or first blood gas base excess (per 10 mmol/L)	–2.620	–4.541	–0.698	**0.008**
Thompson encephalopathy score at admission	0.490	0.152	0.827	**0.005**
Body temperature at admission (°C)	3.742	1.904	5.580	**<0.001**

Statistical significance for univariate and multivariate analysis was assumed for $p < 0.003$ (Bonferroni correction) and $p < 0.05$, respectively (indicated in bold).

[a]Per 10 minutes.

[b]After initiating cooling.
