# Supplementary material for: Body Temperature, Heart Rate, and Short-Term Outcome of Cooled Infants
Source: Ther Hypothermia Temp Manag. 2019 Mar 6;9(1):76–85. doi: 10.1089/ther.2018.0019 (PMC6434598; doi:10.1089/ther.2018.0019)
Supplement: Supplemental data [file Supp_Table3.pdf]

Supplementary Table S3. Control Variables of Heart Rate After 24 Hours of Cooling

	Regression coefficient	*95% CI*		p
		Lower	*Upper*	
(A) Univariate analysis				
Gestational age (weeks)	–1.740	–2.788	–0.693	**0.001**
Birth weight (kg)	0.315	–3.137	3.768	0.858
Birth location (outborn)	3.897	0.335	7.459	0.032
10 minutes Apgar score	–2.448	–3.187	–1.709	**<0.001**
Cord or first blood gas pH (per 0.1 change)	–2.053	–2.799	–1.307	**<0.001**
Cord or first blood gas base excess (per 10 mmol/L)	–4.558	–6.034	–3.083	**<0.001**
Cooling modality (Selective-head)	1.755	–1.865	5.375	0.340
Time of admission after birth[a]	0.093	–0.103	0.289	0.351
Initiating cooling after admission[a]	–0.053	–0.251	0.145	0.596
Initiating cooling after birth[a]	0.024	–0.147	0.195	0.782
Time to reach the target temperature after birth[a]	0.032	–0.052	0.117	0.452
Sarnat encephalopathy stage at admission	10.844	8.205	13.483	**<0.001**
Thompson encephalopathy score at admission	0.963	0.662	1.265	**<0.001**
Thompson encephalopathy score at 24 hours[b]	1.030	0.699	1.360	**<0.001**
Mean blood pressure at 0 hour[b] (per 10 mmHg)	–0.007	–1.864	1.850	0.994
Mean blood pressure at 24 hours[b] (per 10 mmHg)	–0.623	–2.753	1.508	0.566
Body temperature at admission (°C)	–2.103	–3.619	–0.587	0.008
Body temperature at 0 hour[b] (°C)	0.083	–1.357	1.524	0.909
Body temperature at 24 hours[b] (°C)	7.393	4.017	10.769	**<0.001**
Fever ≥38°C from admission to 84 hours[b]	1.658	–11.190	14.506	0.786
(B) Multivariate model				
Cord or first blood gas base excess (per 10 mmol/L)	–3.728	–5.239	–2.216	**<0.001**
Thompson encephalopathy score at admission	0.777	0.484	1.070	**<0.001**
Body temperature at 24 hours[b] (°C)	6.243	2.968	9.517	**<0.001**

Statistical significance for univariate and multivariate analysis was assumed for $p < 0.003$ (Bonferroni correction) and $p < 0.05$, respectively (indicated in bold).

[a]Per 10 minutes.

[b]After initiating cooling.
