# Supplementary material for: Body Temperature, Heart Rate, and Short-Term Outcome of Cooled Infants
Source: Ther Hypothermia Temp Manag. 2019 Mar 6;9(1):76–85. doi: 10.1089/ther.2018.0019 (PMC6434598; doi:10.1089/ther.2018.0019)
Supplement: Supplemental data [file Supp_Table4.pdf]

Supplementary Table S4. Control Variables of Body Temperature at Admission

	Regression coefficient	95% CI Lower	95% CI Upper	p
(A) Univariate analysis				
Gestational age (weeks)	0.093	0.021	0.164	0.011
Birth weight (kg)	0.590	0.330	0.850	**<0.001**
Birth location (outborn)	–0.309	–0.578	–0.039	0.025
10 minutes Apgar score	0.139	0.083	0.195	**<0.001**
Cord or first blood gas pH (per 0.1 change)	0.125	0.060	0.191	**<0.001**
Cord or first blood gas base excess (per 10 mmol/L)	0.265	0.140	0.390	**<0.001**
Time of admission after birth[a]	0.022	0.005	0.040	0.013
Initiating cooling after admission[a]	0.009	–0.006	0.023	0.231
Initiating cooling after birth[a]	0.025	0.013	0.037	**<0.001**
Sarnat encephalopathy stage at admission	–0.561	–0.785	–0.338	**<0.001**
Thompson encephalopathy score at admission	–0.041	–0.067	–0.016	**0.002**
Heart rate at 0 hour[b] (per 10 beat/min)	0.118	0.034	0.202	0.009
Mean blood pressure at 0 hour[b] (per 10 mmHg)	0.173	0.048	0.298	0.007
(B) Multivariate model				
Birth location (outborn)	–0.379	–0.644	–0.114	**0.005**
Birth weight (kg)	0.567	0.310	0.824	**<0.001**
Cord or first blood gas base excess (per 10 mmol/L)	0.207	0.087	0.327	**0.001**
Thompson encephalopathy score at admission	–0.032	–0.058	–0.007	**0.013**

Statistical significance for univariate and multivariate analysis was assumed for $p < 0.004$ (Bonferroni correction) and $p < 0.05$, respectively (indicated in bold).

[a]Per 10 minutes.

[b]After initiating cooling.
