# Supplementary material for: Body Temperature, Heart Rate, and Short-Term Outcome of Cooled Infants
Source: Ther Hypothermia Temp Manag. 2019 Mar 6;9(1):76–85. doi: 10.1089/ther.2018.0019 (PMC6434598; doi:10.1089/ther.2018.0019)
Supplement: Supplemental data [file Supp_Table5.pdf]

Supplementary Table S5. Control Variables of Body Temperature After 24 Hours of Cooling

	Regression coefficient	*95% CI*		p
		Lower	*Upper*	
(A) Univariate analysis				
Gestational age (weeks)	0.006	–0.021	0.033	0.652
Birth weight (kg)	–0.002	–0.097	0.094	0.974
Birth location (outborn)	0.045	–0.059	0.148	0.398
10-minutes Apgar score	–0.035	–0.057	–0.013	**0.002**
Cord or first blood gas pH (per 0.1 change)	–0.015	–0.042	0.013	0.280
Cord or first blood gas base excess (per 10 mmol/L)	–0.018	–0.067	0.031	0.472
Cooling modality (selective-head)	0.409	0.315	0.503	<**0.001**
Time of admission after birth[a]	–0.001	–0.007	0.005	0.815
Initiating cooling after admission[a]	0.010	0.004	0.015	**0.001**
Initiating cooling after birth[a]	0.008	0.003	0.013	**0.001**
Time to reach the target temperature after birth[a]	0.005	0.003	0.008	<**0.001**
Sarnat encephalopathy stage at admission	0.167	0.087	0.247	<**0.001**
Thompson encephalopathy score at admission	0.010	0.001	0.018	0.021
Thompson encephalopathy score at 24 hours[b]	0.009	0.001	0.017	0.034
Heart rate at 0 hour[b] (per 10 beat/min)	0.030	0.002	0.058	0.035
Heart rate at 24 hours[b] (per 10 beat/min)	0.060	0.032	0.089	<**0.001**
Mean blood pressure at 0 hour[b] (per 10 mmHg)	0.008	–0.046	0.062	0.762
Mean blood pressure at 24 hours[b] (per 10 mmHg)	0.050	–0.041	0.140	0.257
Body temperature at admission (°C)	–0.017	–0.054	0.020	0.374
Fever ≥38°C from admission to 84 hours[b]	0.143	–0.156	0.441	0.337
(B) Multivariate model				
10 minutes Apgar score	–0.027	–0.047	–0.007	**0.009**
Cooling modality (selective-head)	0.395	0.301	0.490	<**0.001**

Statistical significance for univariate and multivariate analysis was assumed for $p < 0.003$ (Bonferroni correction) and $p < 0.05$, respectively (indicated in bold).

[a]Per 10 minutes.

[b]After initiating cooling.
